# Supplementary material for: Splice-Junction-Based Mapping of Alternative Isoforms in the Human Proteome
Source: Cell Rep. Author manuscript; Available in PMC 2020 Jan 15. (PMC6961840; doi:10.1016/j.celrep.2019.11.026)
Supplement: 2 [file NIHMS1546469-supplement-2.zip › DF1/PXD006675/RightCoronaryArtery/RightCoronaryArtery_3_MCAM_HLPSLSESQGVPGEAEQPAPELVEVEVGSTALLK.pdf]

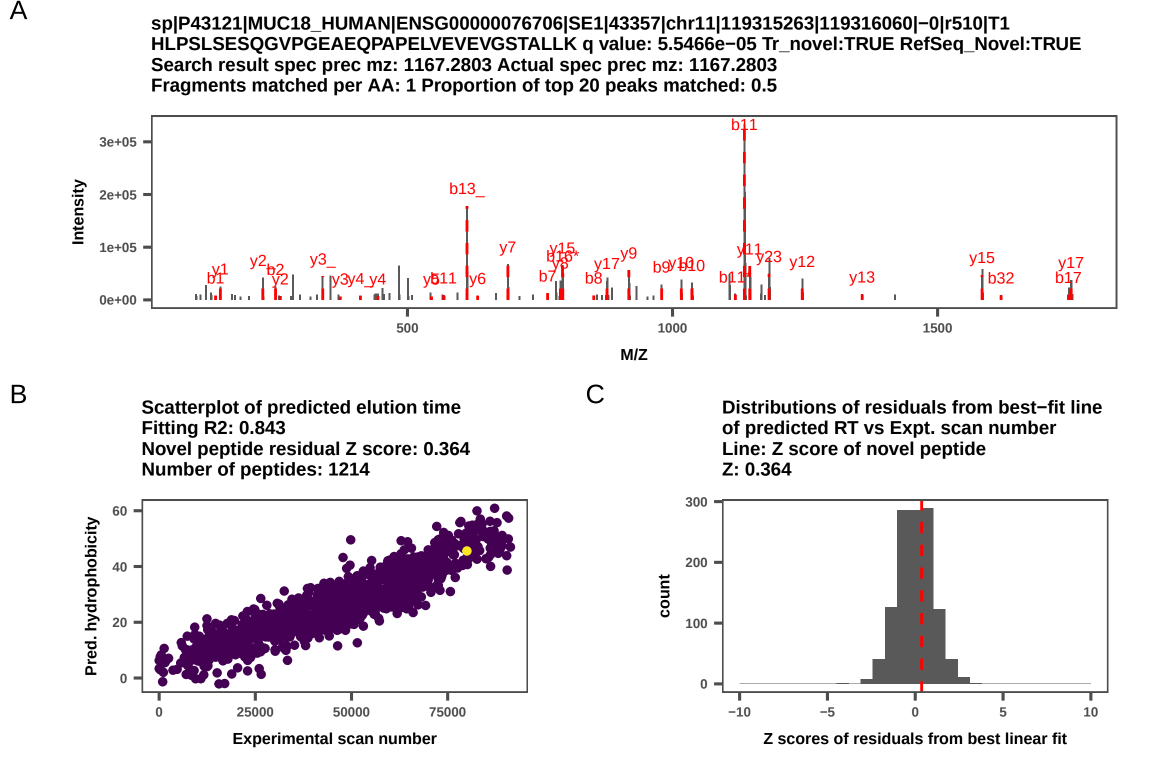

A
sp|P43121|MUC18_HUMAN|ENSG00000076706|SE1|43357|chr11|119315263|119316060|−0|r510|T1
HLPSLSESQGVPGEAEQPAPELVEVEVGSTALLK q value: 5.5466e−05 Tr_novel:TRUE RefSeq_Novel:TRUE
Search result spec prec mz: 1167.2803 Actual spec prec mz: 1167.2803
Fragments matched per AA: 1 Proportion of top 20 peaks matched: 0.5
Intensity
M/Z
B
Scatterplot of predicted elution time
Fitting R2: 0.843
Novel peptide residual Z score: 0.364
Number of peptides: 1214
Pred. hydrophobicity
Experimental scan number
C
Distributions of residuals from best−fit line
of predicted RT vs Expt. scan number
Line: Z score of novel peptide
Z: 0.364
count
Z scores of residuals from best linear fit
